# Prevalence of K13-propeller gene polymorphisms among *Plasmodium falciparum* parasites isolated from adult symptomatic patients in northern Uganda

**DOI:** 10.1186/s12879-016-1777-7

**Published:** 2016-08-19

**Authors:** Moses Ocan, Freddie Bwanga, Alfred Okeng, Fred Katabazi, Edgar Kigozi, Samuel Kyobe, Jasper Ogwal-Okeng, Celestino Obua

**Affiliations:** 1 Department of Pharmacology & Therapeutics, Makerere University, P. O. Box 7072, Kampala, Uganda; 2 Department of Medical Microbiology, Makerere University, P. O. Box 7072, Kampala, Uganda; 3 MBN Clinical Laboratories, P. O. Box 35135, Kampala, Uganda; 4 Lira University, P. O. Box 1035, Lira, Uganda; 5 Mbarara University of Science and Technology, P. O. Box 1410, Mbarara, Uganda

**Keywords:** Artemisinin, Resistance, Medicines, Northern Uganda

## Abstract

**Background:**

In the absence of an effective vaccine, malaria treatment and eradication is still a challenge in most endemic areas globally. This is especially the case with the current reported emergence of resistance to artemisinin agents in Southeast Asia. This study therefore explored the prevalence of K13-propeller gene polymorphisms among *Plasmodium falciparum* parasites in northern Uganda.

**Methods:**

Adult patients (≥18 years) presenting to out-patients department of Lira and Gulu regional referral hospitals in northern Uganda were randomly recruited. Laboratory investigation for presence of plasmodium infection among patients was done using *Plasmodium falciparum* exclusive rapid diagnostic test, histidine rich protein-2 (HRP2) (Pf). Finger prick capillary blood from patients with a positive malaria test was spotted on a filter paper Whatman no. 903. The parasite DNA was extracted using chelex resin method and sequenced for mutations in K13-propeller gene using Sanger sequencing. PCR DNA sequence products were analyzed using in DNAsp 5.10.01software, data was further processed in Excel spreadsheet 2007.

**Results:**

A total of 60 parasite DNA samples were sequenced. Polymorphisms in the K13-propeller gene were detected in four (4) of the 60 parasite DNA samples sequenced. A non-synonymous polymorphism at codon 533 previously detected in Cambodia was found in the parasite DNA samples analyzed. Polymorphisms at codon 522 (non-synonymous) and codon 509 (synonymous) were also found in the samples analyzed. The study found evidence of positive selection in the *Plasmodium falciparum* population in northern Uganda (Tajima’s D = −1.83205; Fu and Li’s D = −1.82458).

**Conclusions:**

Polymorphism in the K13-propeller gene previously reported in Cambodia has been found in the Ugandan *Plasmodium falciparum* parasites. There is need for continuous surveillance for artemisinin resistance gene markers in the country.

**Electronic supplementary material:**

The online version of this article (doi:10.1186/s12879-016-1777-7) contains supplementary material, which is available to authorized users.

## Background

Malaria is the leading cause of morbidity and mortality in Uganda accounting for 30–50 % of outpatient visits, 15–20 % of hospital admissions and is responsible for nearly half of all inpatient pediatric deaths [1]. Uganda has the third highest number of malaria deaths in Africa in addition to having the highest reported malaria transmission rates in the world [2]. The country also has a high (90–95 %), stable and perennial malaria transmission throughout the year [3]. In Uganda, national policy for treatment of uncomplicated malaria was first changed from chloroquine (CQ) monotherapy to CQ plus sulfadoxine-pyrimethamine (SP) (CQ + SP) combination therapy in 2000. However this was also changed to the current artemisinin based combination (ACTs) antimalarial agents in 2004 due to wide spread resistance to the earlier agents [4].

The artemisinin agents are rapidly acting and significantly reduce the biomass of sensitive parasites corresponding to a single cycle of a sexual blood stage of *Plasmodium falciparum* in 48 h [5]. The use of short acting artemisinin (half-life <1 h) and long-lasting partner drug (lumefantrine, amodiaquine, or piperaquine) in artemisinin-based combination therapy (ACTs), has contributed to an estimated 30 % reduction in global rate of malaria associated mortality in the past decade [6]. However, the emergence of artemisinin resistance in Southeast Asia threatens malaria control and prevention programs globally [7]. With the experience of chloroquine resistance in which it resulted in doubling of malaria associated mortality in sub-Saharan Africa [8], the emergence of artemisinin resistance [9] could potentially pose a similar risk especially in malaria endemic areas of the world.

Artemisinin resistance is currently associated with polymorphisms in the K13-propeller gene [9]. Genomic analysis of Cambodian parasite isolates have identified four prevalent K13-propeller mutations (Y493H, R539T, I543T, and C580Y) that are associated with elevated ring-stage survival rates In vitro and long parasite clearance half-lives (>5 h) [10]. Evidence from previous studies have indicated that artemisinin resistance affects early ring-stage of the plasmodium parasites’ intraerythrocytic developmental cycle [11].

The spread of artemisinin resistance to other parts of the world is quite likely as previous parasite resistance to chloroquine and sulphadoxine-pyrimethamine all arose from the same region, Cambodia and spread globally [12, 13]. This is further indicated by the current spread of artemisinin resistance in most provinces of Cambodia, Thailand-Myanmar border, southern Vietnam, southern Laos and central Myanmar [14]. It thus seems a matter of time before the K13 gene polymorphisms detected in Cambodia and associated with artemisinin resistance can spread to parts of the world outside Southeast Asia. With limited or no current alternative medicines to artemisinin agents in addition to lack of an effective vaccine, the future of malaria treatment is facing perhaps the greatest test since the emergence of chloroquine resistance more than a decade ago.

With increased use of artemisinin based combination agents in malaria treatment globally [2], surveillance programs are needed to help monitor the emergence and spread of resistance. This study was thus intended to investigate the prevalence of resistance gene markers to artemisinin antimalarial agents among *Plasmodium falciparum* parasites isolated from adult patients (≥18 years) reporting to hospitals in northern Uganda.

## Methods

### Study design, area and population

**Fig. 1:**
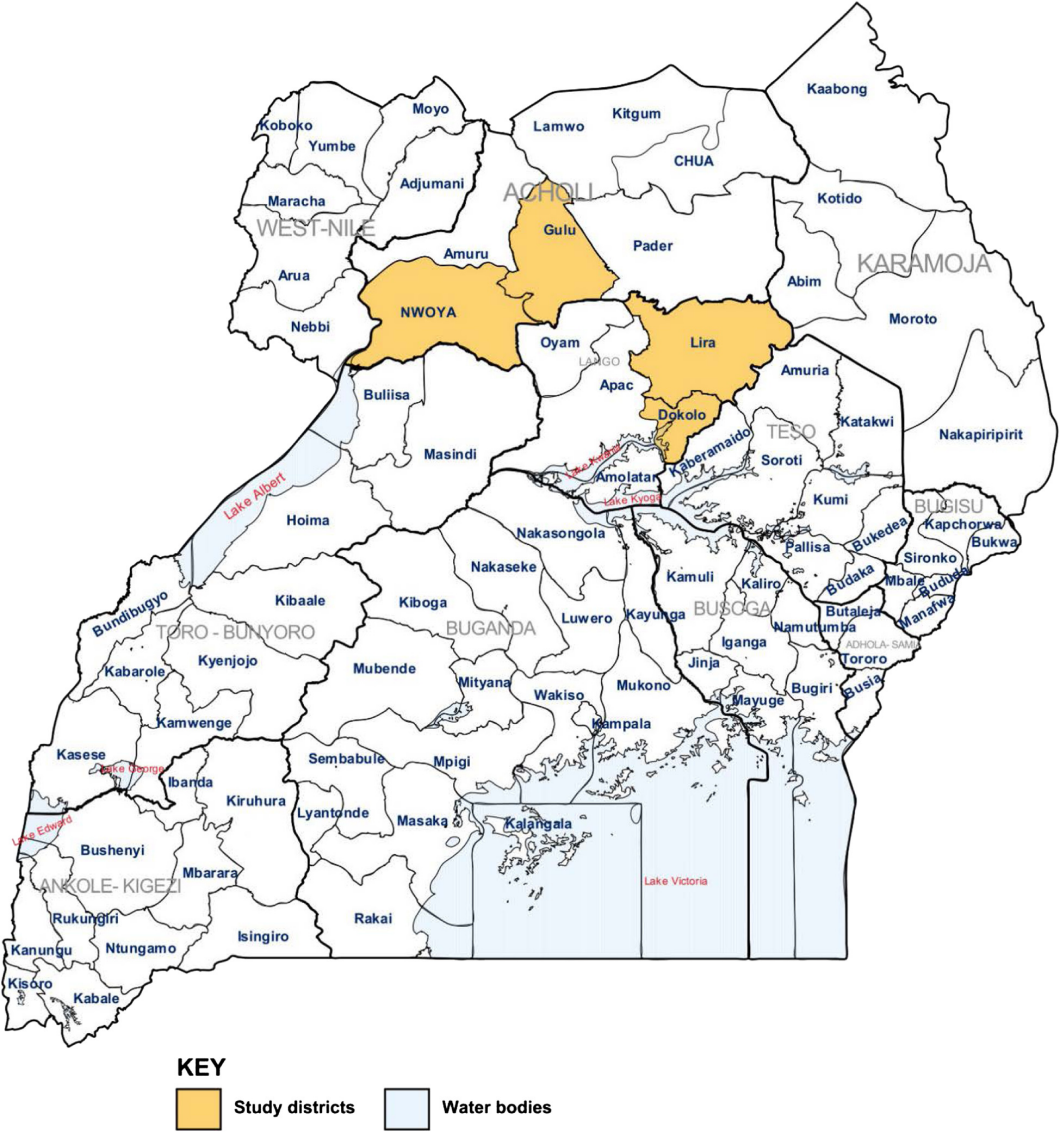
Map of Uganda showing the districts where the study was conducted in northern Uganda: *An updated map of Uganda as provided by the United Nations Office for the Coordination of Humanitarian Affairs, 2006; based on OCHA/ReliefWeb*

This was a cross sectional study done among patients presenting with symptoms of malaria (fever) to Lira and Gulu regional referral hospitals (RRHs) in northern Uganda. The two hospitals are more than 300 km from the capital Kampala and the districts of their location are shown in the map of Uganda, (Fig. 1).

### Sampling criteria

Adult patients (≥18 years) reporting to the general laboratory in out-patients department (OPD) with a request from the doctor for a malaria test were all contacted for recruitment into the study using the interval of 4 and 3 in Lira and Gulu regional referral hospitals respectively. Patients who provided written informed consent were then recruited into the study.

### Laboratory analysis

#### Sample collection and processing

Data collection was done from August 2013 to May 2014 with blood sample collection occurring between 8 AM -to- 4 PM on each working day (Monday-to-Friday) during the study period. Capillary blood from a finger prick was collected from each patient after sterilizing the fingertip using alcohol swab. The blood was then tested for presence of plasmodium infection using a *Plasmodium falciparum* exclusive rapid diagnostic test, HRP2 (Pf) (Access Bio, Inc, USA). The patients with positive result for *Plasmodium falciparum* infection were notified and more capillary blood collected and spotted on a filter paper Whatman No. 903. Plasmodium parasite DNA was extracted from the dried blood spots on filter paper using chelex resin method as previously described by Plowe, 1995 [15]. Polymorphisms at K13-propeller gene of *Plasmodium falciparum* parasite was determined using Sanger sequencing. This was done in the molecular diagnostic laboratory (MBN Clinical Laboratories Ltd), P. O. Box 35135, Kampala, Uganda (www.mbnlab.com).

### PCR amplification and sequencing of *P. falciparum* isolates

For amplification of the K13 gene, we used nested PCR to amplify 849 bp fragment extending from nucleotides 1329-to-2178 (codons 445-to-727) within the entire K13-propeller domain. The first and second round amplification reactions were performed using a Thermal cycler (Bio-Rad, T100; Singapore) in 25 and 50 μl reaction volumes respectively. The K13-propeller domain was amplified using the following primers (Eurofins Genomics, German);

First round PCR (K131-5′CGGAGTGACCAAATCTGGGA-3′ and

K134-5′-GGGAATCTGGTGGTAACAGC-3′),

Second round PCR (K133-5′-GCCTTGTTGAAAGAAGCAGA-3′;

K132-5′GCCAAGCTGCCATTCATTTG-3′).

First round PCR was run in a 25 μl reaction volume: briefly, 5.5 μl of nuclease free water (QIAGEN, Maryland, USA) was added to 12.5 μl of *Taq* 2X Master Mix (New England BioLabs, Massachusetts, USA). We used *Taq* 2X Master Mix with the final working concentration of the components at 1X. The concentration of the components at 1X were: 10 mM Tris-HCl, 50 mM KCl, 1.5 mM MgCl_2_, 0.2 mM dNTPs, 25 units/ml *Taq* DNA Polymerase, 5 % Glycerol, 0.08 % IGEPAL® CA-630, 0.05 % Tween®, pH 8.6@25 °C). To this 1.0 μl of 10pmol/μl each of the forward and reverse primers was added. 5.0 μl of 50 ng/μl DNA template was then added. The PCR was run using the profile that included Initial denaturation at 94 °C for 5 m, followed by 40 cycles of Denaturation at 94 °C for 30s, Primer annealing at 54 °C for 90s and Extension at 68 °C for 90s. Final extension was then done at 68 °C for 10 min.

The products of the first round PCR were used as templates for the second round PCR. This was run in a 50 μl reaction volume; briefly, 19 μl of nuclease free water (QIAGEN, Maryland, USA) was added to 25 μl of *Taq* 2X Master Mix (New England BioLabs, Massachusetts, USA). The final working concentrations of the components of the Master Mix for the second round PCR were similar to those of the first round PCR. 2.0 μl of 10pmol/μl each of the forward and reverse primers was added. To this mixture was then added 2.0 μl of the first round PCR amplicons and the PCR reaction run using the amplification profile as described above.

All PCR products were analyzed using 1 % (w/v) Agarose gel (Fisher Scientific, USA), stained with 0.05 μg/mL of Ethidium Bromide (Sigma Aldrich, USA).

### Sequencing of K13 propeller gene

To sequence the K13 propeller gene, the second round PCR products were purified using jet quick PCR product purification kit (Genomed, Cat number 41-0050). 5 μl of the purified second round PCR products were run on 1.0 % (w/v) Agarose gel stained with 0.05 μg/mL of Ethidium Bromide (Sigma Aldrich, USA) to check for the presence and concentration of PCR products. Bi-directional cycle sequencing of K13 was done using second round PCR primers K133 Forward and K132 Reverse (Eurofins Genomics, Germany), using the BigDye terminator v3.1 cycle sequencing kit (Applied Bio systems, USA). The cycle sequencing PCR was performed in a total reaction volume of 20 μl. Briefly, 6.0 μl of the BigDye terminator v3.1 5X sequencing buffer (Applied Bio systems, USA) was aliquoted and mixed with; 2.0 μl BigDye terminator v3.1 (Applied Bio systems, USA), 1.0ul of 10 ng/μl (K133Fwd or K132Rev) primers and 6.0ul of nuclease free water. Then, 5.0ul of 5.0 ng/μl of the purified second round PCR products was added. The sequencing reactions where run in the Thermal cycler (Gene Amp 9700 PCR system, USA) under the following conditions. One cycle of Polymerase activation at 96 °C for 60s followed by 35 cycles of; denaturation at 96 °C for 10s, annealing at 53 °C for 30s, extension at 60 °C for 300 s. The products were then stored at 4 °C until the next step. The Extension (Cycle sequencing) PCR products were then purified using DyeEx 2.0 spin Kit (QIAGEN, Maryland, USA). 5.0 μl of the purified cycle sequencing PCR products were pipetted and mixed with 5.0 μl of De-ionized formamide (Sigma Aldrich, USA). This mixture was then loaded in the 310 genetic analyzer (Applied Bio systems, USA) and the products bi-directional sequenced with POP-7™ (Applied Bio systems, USA) as a sequencing Polymer.

### Sequence data analysis

The sequence data were base called using the sequence analysis software 5.2 and then blasted on to the NCBI sequence data base to confirm the K13 propeller gene sequences identity. The sequences were imported in to bio edit sequence alignment editor 7.2.5 for manual editing and then exported in to MEGA 5 software version 5.10 for detection of polymorphism using the *PF3D7_1343700* K13-propeller gene sequences in NCBI database as the reference sequence. Further SNPs analysis within the K13 propeller gene was performed using the DnaSP software version 5.10.01.

We assessed the selection pressure in *Plasmodium falciparum* parasite population in northern Uganda using Tajima’ D statistic and Fu & Li’s D test in DnaSP software 5.10.01. In this analysis we evaluated whether the *P. falciparum* K13 propeller domain sequence data shows evidence of deviation from neutrality theory of molecular evolution [16]. A total of 60 (sixty) DNA sample sequence data of *P. falciparum* parasites from Lira and Gulu districts were exported directly from Bioedit into the DnaSP software for computation of Tajima’s D and Fu & Li’s D statistic. The analysis was done using commands in the DnaSP software. In DnaSP software, the probability of Tajima’s D and Fu & Li’s D are estimated by simulation. The test uses information on frequency of mutations (allelic variation) [17]. Tajima’s D and Fu & Li’s D test is based on the fact that under the neutral model, estimates of the number of polymorphic sites and of the average number of nucleotide differences are correlated. The critical values (Tajima’s D and Fu & Li’s D) obtained were used in interpreting the findings under neutrality assumption [16, 18].

### Tajima’s D simulation

$$ D=\frac{\pi -\raisebox{1ex}{$S$}\!\left/ \!\raisebox{-1ex}{${a}_1$}\right.}{\sqrt{Var\left[\pi -\raisebox{1ex}{$S$}\!\left/ \!\raisebox{-1ex}{${a}_1$}\right.\right]}} $$

Where,

*π* = Mean pairwise differences

*S* = Number of segregating sites

α_1_ = $$ {\displaystyle {\sum}_{i=1}^{n=1}\raisebox{1ex}{$1$}\!\left/ \!\raisebox{-1ex}{$i$}\right.} $$

### Fu and Li’s D simulation

$$ D=\frac{S-{a}_{1\cap e}}{\sqrt{Var\left(S-{a}_1\cap e\right)}} $$

Where,

∩ *e* = Expected number of derived mutations that are present only once in the sample

*S* = Number of segregating sites

*a*_1_ = $$ {\displaystyle {\sum}_{i=1}^{n=1}\raisebox{1ex}{$1$}\!\left/ \!\raisebox{-1ex}{$i$}\right.} $$

### Statistics and analysis

Data was analyzed in Excel spread sheet 2007.

## Results

We collected blood samples from a total of 100 patients with symptomatic *Plasmodium falciparum* infection (malaria) who visited out-patient departments of Lira and Gulu regional referral hospitals (50 patients from each hospital). The average age of the study participants was 28 ± 8.7. The study was conducted in the outpatient department laboratory of Lira and Gulu regional referral hospitals in northern Uganda. Sixty (60) parasite DNA samples that successfully amplified in the nested PCR reaction for K13-propeller gene were all sequenced.

### Prevalence of K13 polymorphisms

Single nucleotide polymorphisms (SNPs) found in the K13-propeller gene include; 509, 522, and 533 (Table 1 and Additional file 1). One of the nonsynonymous SNPs (533) found in the current study has been previously reported in Cambodia [9]. Mutation at codon 509 was found in two samples while others were present in one sample each.

**Table 1:** Single nucleotide substitutions in K13 gene among the *P. falciparum* samples

Mutant nucleotide position	Mutant codon position	Reference codon	Mutant codon	Transition	Reference amino acid	Mutant amino acid	Type of mutation	Prevalence of the mutation % (n)
1527	509	GAG	GAA	G→A	Glutamine	Glutamine	S	3.3 % (*n* = 2)
1566	522	AGT	AGG	T→G	Serine	Arginine	NS	1.7 % (*n* = 1)
1599	533	GGT	TGT	G→T	Glycine	Cysteine	NS	1.7 % (*n* = 1)

*NS* non-synonymous mutation, *S* synonymous mutation

In total, there were four (4) polymorphic sites identified in the 60 samples analyzed. The mutations in the samples analyzed were not selectively neutral as shown by the negative Tajima’s D statistic (−1.83205) and Fu & Li’s D test (−1.82458). There was no significant haplotype/gene diversity (*P* = 0.215) with the variance in diversity of 0.0056 and the standard deviation of 0.075 (Table 2).

**Table 2:** Haplotype diversity of K13 propeller domain mutation in the DNA analyzed

K13 analysis parameters	Lira	Gulu	Overall
Sample size	38	22	60
Number of Haplotypes (h)	2	2	4
Haplotype (gene) diversity (Hd)	0.201	0.242	0.215
Variance of Haplotype diversity	0.00728	0.01831	0.00564
Standard Deviation of Haplotype diversity	0.085	0.135	0.075
Number of nucleotide sites analyzed	587	587	587
Nucleotide diversity	0.00287	0.00119	0.00132
Tajima’s D statistic	−0.88600	−2.27026	−1.83205 (Not significant; *P* <0.05)
Fu and Li’s D test statistic	−0.33513	−1.9147	−1.82458 (Not significant; *P* <0.05)
Number of polymorphic sites	2	2	4

## Discussion

Resistance to the artemisinin agents can be assessed using both molecular (genotypic) and phenotypic (Ring Stage survival Assay; RSA_0–3hr_) methods. Artemisinin resistance mainly manifests as delayed parasite clearance and is associated with decreased In vitro action of pulses of artemisinin agents [19, 20] that is associated with ACT failures [21]. In this study we used molecular parasite characterization to denote existence or absence of resistance to artemisinin agents among the *Plasmodium falciparum* parasite population in northern Uganda.

In the current study, non-synonymous mutations at codon 522 and 533 of the K13 propeller gene were detected in the *Plasmodium falciparum* parasites. The mutation at codon 533 found in the current study has previously been reported in *Plasmodium falciparum* parasites in Cambodia [9]. The parasites carrying this mutation were assessed for phenotypic resistance using RSA _0–3h_ method, and were shown not to be associated with artemisinin resistance in a study by Ariey et al.*,* [22]. A study by Feng et al.*,* [23] reported presence of polymorphism at codons 537 and 574 of the K13 propeller gene in *Plasmodium falciparum* parasites isolated from Ghanaian migrants to China, mutations previously reported in Cambodia [22]. A mutation at codon 522 found in our study was also reported in a previous study done in Uganda by Conrad et al.*,* [24]. However whether these mutations are associated with *Plasmodium falciparum* parasite artemisinin resistance has not been demonstrated. Recent studies in Uganda have shown artemisinin agents to be effective in clearing parasites in malaria treatment [25, 26]. A study by Cooper et al.*,* [27] done in Uganda failed to demonstrate in an ex vivo assay using RSA_0–3hr_ method the presence of decreased sensitivity of *Plasmodium falciparum* parasite isolates to the artemisinin agents.

The polymorphisms in K13-propeller gene, C580Y, R539T, R543I and Y493H reported in Cambodia and associated with artemisinin resistance [9] were not found in the current study. Our findings in addition to those of Feng et al.*,* [23] done in Ghana, and other African countries [28] are indicative of the potential risk of emergence of artemisinin resistance among *Plasmodium falciparum* parasites in Sub-Saharan Africa. One mutation at codon position 509 found in our study has not previously been reported by other studies. Recent studies done in Uganda [24, 27], Africa [28–30], Myanmar [31] have also found mutations in malaria parasites not previously reported in Cambodia [22]. Such geographical variations in the K13-propeller gene mutations is indicative of the existence of a reservoir for the K13-propeller gene polymorphisms globally [28]. However whether this mutations are correlated with reduced parasite clearance in malaria treatment by the artemisinin agents needs to be elucidated as this would help in tracking artemisinin resistance in malaria endemic areas outside of Southeast Asia.

Due to high malaria transmission in sub-Saharan Africa, it is likely that this could result in high use of artemisinin agents. In Uganda, a study by Ocan et al.*,* [32] found high prevalence, 29 % of non-prescription use of ACTs (Coartem) in northern Uganda. Uganda adopted a policy on home based treatment of fever using artemisinin combination agents [33] and presumptive treatment of all fevers as malaria [34]. A report by world health organization [2], also showed a 30-fold increase in the use of ACTs globally between 2006 and 2013. This increased use of artemisinin agents is likely to raise drug pressure and hence the risk of resistance development [35]. In addition, inappropriate use of artemisinin antimalarial agents coupled with the risk of using medicines of substandard quality common in developing countries may further exacerbate the risk of resistance development [2]. Like chloroquine and sulfadoxine-pyrimethamine resistance which first emerged in Southeast Asia then spread to other parts of the world [36], it is possible that the artemisinin resistance that has been detected in Cambodia could also spread through Myanmar via India to Africa [37]. This is likely due to the increased international travel and migration [38]. When we tested the departures of nucleotide variability patterns of the sequence products from neutral expectations using Tajima’s D and Fu & Li’s D test statistic, there was evidence of positive selection (or selective sweeps) as indicated by the negative values of the tests (Tajima’s D = −1.83205; Fu and Li’s D of −1.82458) [39]. Previous studies have shown that local ecological and population level processes such as drug pressure are important drivers of resistance development in the population [38–40]. However this study could not establish whether the K13-propeller gene mutations observed in the *Plasmodium falciparum* parasite population in northern Uganda were as a result of transfer through global human travel or local emergence as a result of ecological and population level processes.

Unlike Africa where Artemisinin agents are mostly used in combinations, studies have shown that up to 78 % of their use in Cambodia are as monotherapy [41]. The basis for artemisinin combination therapy (ACTs) is that the artemisinin component would rapidly reduce parasite load in blood, quickly resolve symptoms, and the partner drug would remain active for days or weeks and ‘mop up’ any remaining parasites [5]. The use of artemisinin agents in combination coupled with their short half-lives may still be protecting the population from wide spread emergence of resistant parasites [42]. However, Uganda recently also adopted the use of artemisinin monotherapy (IV artesunate) in treatment of complicated malaria [43]. The use of Artemisinin agents especially as monotherapy has been associated with selective pressure in parasites isolated from French Guyana and Senegal [44]. A review by Winzeler, [41] also reported failure of parasite clearance among patients treated with artesunate monotherapy. Therefore use of artemisinin monotherapy could potentially enhance the risk of resistance development in the country.

Artemisinin agents are used in combination and therefore when resistance develops to the artemisinin component the other partner drug could be sufficiently effective in order for the combination to retain its effectiveness. However, studies have also reported existence of resistance to the partner agents commonly used in artemisinin combination therapy; amodiaquine, lumefantrine, and mefloquine [45, 46]. Therefore development of resistance to the artemisinin component is a potential threat to the antimalarial artemisinin combination therapy. Studies in Uganda have not been able to demonstrate failure of parasite clearance by the artemisinin combination agents used in malaria treatment [24, 26]. However there are reports of decreasing malaria treatment cure rates by the artemisinin combination antimalarial agents in Africa [47] and Asia [48]. Phenotypic and genotypic findings of previous studies [24, 27] coupled with those of our current study in addition to the prevalent inappropriate use of ACTs in communities [49], support the need for regular epidemiological surveillance of resistance to artemisinin agents and the partner drugs currently used in malaria treatment in Uganda.

Currently there is no known alternative medicine to artemisinin based antimalarial agents for malaria treatment globally. Therefore the possibility of emergence of artemisinin resistance is likely to increase the risk of morbidity and mortality from malaria especially in endemic areas of the world. Unfortunately there is no tangible response to the likelihood of artemisinin resistance development in most malaria endemic countries globally. This is indicated by the findings of our current study in which ACTs (Coartem) are easily accessed and used without prescription in the communities [32]. This could further exacerbate the risk of resistance development in the country.

In most parts of the world other than South East Asia, artemisinin combination agents remain very effective antimalarial drugs [50]. In Uganda delayed parasite clearance of ACTs has not yet been detected in malaria treatment [51]. Partial host immunity from repeated exposure due to high malaria transmission common in sub-Saharan Africa may be masking the loss of efficacy to ACTs [52]. A study in Mali showed a correlation between immunity against malaria infection among patient and fast rates of artemisinin-induced parasite clearance [53].

Due to either the low parasite load as we did not quantify parasitaemia during blood sample collection or poor DNA quality, we could not successfully amplify *Plasmodium falciparum* K13-propeller gene from all the 100 extracted parasite DNA samples.

## Conclusion

This study showed existence of K13-propeller gene mutations among *Plasmodium falciparum* parasite population in northern Uganda. However the polymorphisms associated with delayed *Plasmodium* parasite clearance by artemisinin agents previously reported in Cambodia were not found in our study. Regular epidemiological surveillance of molecular and phenotypic artemisinin resistance in the country is thus needed. This will help in tracking the possible development of artemisinin resistance and could help inform updating of malaria treatment guidelines.

## Additional file

Additional file 1: Nucleotide sequences of *Plasmodium falciparum* DNA samples with mutations. This file contains the nucleotide sequences of the *Plasmodium falciparum* parasite DNA samples that were found to have polymorphisms and the wild type strains. (PDF 88 kb)

## Abbreviations

ACT: Artemisinin combination therapy
ART: Artemisinin therapy
DNA: Deoxyribonucleic acid
dNTP: Deoxyribonucleotide triphosphate
HRP: Histidine rich protein
OPD: Out-Patient Department
PCR: Polymerase chain reaction
RRH: Regional referral hospital
SNP: Single nucleotide polymorphism
