# Supplementary material for: Prevalence of K13-propeller gene polymorphisms among Plasmodium falciparum parasites isolated from adult symptomatic patients in northern Uganda
Source: BMC Infect Dis. 2016 Aug 19;16:428. doi: 10.1186/s12879-016-1777-7 (PMC4992308; doi:10.1186/s12879-016-1777-7)
Supplement: Additional file 1: — Nucleotide sequences of Plasmodium falciparum DNA samples with mutations. This file contains the nucleotide sequences of the Plasmodium falciparum parasite DNA samples that were found to have polymorphisms and the wild type strains. (PDF 88 kb) [file 12879_2016_1777_MOESM1_ESM.pdf]

NUCLEOTIDE SEQUENCES OF THE FOUR SAMPLES WITH POLYMORPHISMS

>K13D7K13_WILD TYPE

```
1      ATG GAA GGA GAA AAA GTA AAA ACA AAA GCA AAT AGT ATC TCG AAT   45
1      Met Glu Gly Glu Lys Val Lys Thr Lys Ala Asn Ser Ile Ser Asn   15

46     TTT TCT ATG ACG TAT GAT AGG GAA TCT GGT GGT AAC AGC AAT AGT   90
16     Phe Ser Met Thr Tyr Asp Arg Glu Ser Gly Gly Asn Ser Asn Ser   30

91     GAT GAT AAA AGC GGA AGT AGT AGC GAG AAT GAT TCT AAT TCA TTT   135
31     Asp Asp Lys Ser Gly Ser Ser Ser Glu Asn Asp Ser Asn Ser Phe   45

136    ATG AAT CTA ACT AGT GAT AAA AAT GAG AAA ACG GAA AAT AAT AGT   180
46     Met Asn Leu Thr Ser Asp Lys Asn Glu Lys Thr Glu Asn Asn Ser   60

181    TTC CTT TTA AAT AAT AGT AGT TAT GGA AAT GTT AAA GAT AGC CTA   225
61     Phe Leu Leu Asn Asn Ser Ser Tyr Gly Asn Val Lys Asp Ser Leu   75

226    TTA GAA TCC ATT GAT ATG AGT GTA TTA GAT TCG AAC TTT GAT AGT   270
76     Leu Glu Ser Ile Asp Met Ser Val Leu Asp Ser Asn Phe Asp Ser   90

271    AAA AAA GAT TTT TTA CCA AGT AAT TTA TCA AGA ACA TTT AAT AAT   315
91     Lys Lys Asp Phe Leu Pro Ser Asn Leu Ser Arg Thr Phe Asn Asn   105

316    ATG TCT AAA GAT AAT ATA GGA AAT AAA TAT TTA AAT AAA TTG TTA   360
106    Met Ser Lys Asp Asn Ile Gly Asn Lys Tyr Leu Asn Lys Leu Leu   120

361    AAT AAA AAA AAA GAT ACT ATT ACA AAT GAA AAT AAT AAT ATT AAT   405
121    Asn Lys Lys Lys Asp Thr Ile Thr Asn Glu Asn Asn Asn Ile Asn   135

406    CAT AAT AAT AAT AAT AAT AAT CTG ACA GCA AAT AAT ATA ACT AAT   450
136    His Asn Asn Asn Asn Asn Asn Leu Thr Ala Asn Asn Ile Thr Asn   150

451    AAT CTT ATT AAT AAT AAT ATG AAT TCT CCA TCA ATT ATG AAT ACC   495
151    Asn Leu Ile Asn Asn Asn Met Asn Ser Pro Ser Ile Met Asn Thr   165

496    AAC AAA AAA GAG AAT TTT TTA GAT GCA GCA AAT CTT ATA AAT GAT   540
166    Asn Lys Lys Glu Asn Phe Leu Asp Ala Ala Asn Leu Ile Asn Asp   180

541    GAT TCT GGA TTA AAC AAT TTA AAA AAA TTT TCA ACT GTA AAT AAT   585
181    Asp Ser Gly Leu Asn Asn Leu Lys Lys Phe Ser Thr Val Asn Asn   195

586    GTA AAT GAT ACT TAT GAA AAG AAA ATT ATT GAA ACG GAA TTA AGT   630
196    Val Asn Asp Thr Tyr Glu Lys Lys Ile Ile Glu Thr Glu Leu Ser   210

631    GAT GCT AGT GAT TTT GAA AAT ATG GTA GGT GAT TTA AGA ATT ACA   675
211    Asp Ala Ser Asp Phe Glu Asn Met Val Gly Asp Leu Arg Ile Thr   225

676    TTT ATT AAT TGG TTA AAA AAG ACA CAA ATG AAT TTT ATT CGA GAA   720
```

226 Phe Ile Asn Trp Leu Lys Lys Thr Gln Met Asn Phe Ile Arg Glu 240

721 AAA GAT AAA TTA TTT AAA GAT AAG AAA GAA CTA GAA ATG GAA AGA 765
241 Lys Asp Lys Leu Phe Lys Asp Lys Lys Glu Leu Glu Met Glu Arg 255

766 GTA CGA TTG TAC AAA GAA TTA GAA AAC CGT AAA AAT ATT GAA GAA 810
256 Val Arg Leu Tyr Lys Glu Leu Glu Asn Arg Lys Asn Ile Glu Glu 270

811 CAG AAA TTA CAT GAT GAA AGA AAG AAA TTA GAT ATT GAT ATA TCT 855
271 Gln Lys Leu His Asp Glu Arg Lys Lys Leu Asp Ile Asp Ile Ser 285

856 AAT GGT TAT AAA CAA ATA AAA AAA GAA AAA GAA GAA CAT AGG AAA 900
286 Asn Gly Tyr Lys Gln Ile Lys Lys Glu Lys Glu Glu His Arg Lys 300

901 CGA TTT GAT GAA GAA AGA TTA AGA TTT TTA CAA GAA ATC GAT AAA 945
301 Arg Phe Asp Glu Glu Arg Leu Arg Phe Leu Gln Glu Ile Asp Lys 315

946 ATT AAA TTA GTA TTA TAT TTA GAA AAA GAA AAA TAT TAT CAA GAA 990
316 Ile Lys Leu Val Leu Tyr Leu Glu Lys Glu Lys Tyr Tyr Gln Glu 330

991 TAT AAA AAT TTT GAG AAT GAT AAA AAA AAA ATT GTT GAT GCA AAT 1035
331 Tyr Lys Asn Phe Glu Asn Asp Lys Lys Lys Ile Val Asp Ala Asn 345

1036 ATT GCT ACT GAA ACT ATG ATT GAT ATT AAT GTT GGT GGA GCT ATT 1080
346 Ile Ala Thr Glu Thr Met Ile Asp Ile Asn Val Gly Gly Ala Ile 360

1081 TTT GAA ACA TCT AGA CAT ACC TTA ACA CAA CAA AAA GAT TCA TTT 1125
361 Phe Glu Thr Ser Arg His Thr Leu Thr Gln Gln Lys Asp Ser Phe 375

1126 ATA GAG AAA TTA TTA AGT GGA AGA CAT CAT GTA ACC AGA GAT AAA 1170
376 Ile Glu Lys Leu Leu Ser Gly Arg His His Val Thr Arg Asp Lys 390

1171 CAA GGA AGA ATA TTC TTA GAT AGG GAT AGT GAG TTA TTT AGA ATT 1215
391 Gln Gly Arg Ile Phe Leu Asp Arg Asp Ser Glu Leu Phe Arg Ile 405

1216 ATA CTT AAC TTC TTA AGA AAT CCG TTA ACT ATA CCC ATA CCA AAA 1260
406 Ile Leu Asn Phe Leu Arg Asn Pro Leu Thr Ile Pro Ile Pro Lys 420

1261 GAT TTA AGT GAA AGT GAA GCC TTG TTG AAA GAA GCA GAA TTT TAT 1305
421 Asp Leu Ser Glu Ser Glu Ala Leu Leu Lys Glu Ala Glu Phe Tyr 435

1306 GGT ATT AAA TTT TTA CCA TTC CCA TTA GTA TTT TGT ATA GGT GGA 1350
436 Gly Ile Lys Phe Leu Pro Phe Pro Leu Val Phe Cys Ile Gly Gly 450

1351 TTT GAT GGT GTA GAA TAT TTA AAT TCG ATG GAA TTA TTA GAT ATT 1395
451 Phe Asp Gly Val Glu Tyr Leu Asn Ser Met Glu Leu Leu Asp Ile 465

1396 AGT CAA CAA TGC TGG CGT ATG TGT ACA CCT ATG TCT ACC AAA AAA 1440
466 Ser Gln Gln Cys Trp Arg Met Cys Thr Pro Met Ser Thr Lys Lys 480

1441 GCT TAT TTT GGA AGT GCT GTA TTG AAT AAT TTC TTA TAC GTT TTT 1485
481 Ala Tyr Phe Gly Ser Ala Val Leu Asn Asn Phe Leu Tyr Val Phe 495

1486 GGT GGT AAT AAC TAT GAT TAT AAG GCT TTA TTT GAA ACT GAG GTG 1530
496 Gly Gly Asn Asn Tyr Asp Tyr Lys Ala Leu Phe Glu Thr Glu Val 510

1531 TAT GAT CGT TTA AGA GAT GTA TGG TAT GTT TCA AGT AAT TTA AAT 1575
511 Tyr Asp Arg Leu Arg Asp Val Trp Tyr Val Ser Ser Asn Leu Asn 525

1576 ATA CCT AGA AGA AAT AAT TGT GGT GTT ACG TCA AAT GGT AGA ATT 1620
526 Ile Pro Arg Arg Asn Asn Cys Gly Val Thr Ser Asn Gly Arg Ile 540

1621 TAT TGT ATT GGG GGA TAT GAT GGC TCT TCT ATT ATA CCG AAT GTA 1665
541 Tyr Cys Ile Gly Gly Tyr Asp Gly Ser Ser Ile Ile Pro Asn Val 555

1666 GAA GCA TAT GAT CAT CGT ATG AAA GCA TGG GTA GAG GTG GCA CCT 1710
556 Glu Ala Tyr Asp His Arg Met Lys Ala Trp Val Glu Val Ala Pro 570

1711 TTG AAT ACC CCT AGA TCA TCA GCT ATG TGT GTT GCT TTT GAT AAT 1755
571 Leu Asn Thr Pro Arg Ser Ser Ala Met Cys Val Ala Phe Asp Asn 585

1756 AAA ATT TAT GTC ATT GGT GGA ACT AAT GGT GAG AGA TTA AAT TCT 1800
586 Lys Ile Tyr Val Ile Gly Gly Thr Asn Gly Glu Arg Leu Asn Ser 600

1801 ATT GAA GTA TAT GAA GAA AAA ATG AAT AAA TGG GAA CAA TTT CCA 1845
601 Ile Glu Val Tyr Glu Glu Lys Met Asn Lys Trp Glu Gln Phe Pro 615

1846 TAT GCC TTA TTA GAA GCT AGA AGT TCA GGA GCA GCT TTT AAT TAC 1890
616 Tyr Ala Leu Leu Glu Ala Arg Ser Ser Gly Ala Ala Phe Asn Tyr 630

1891 CTT AAT CAA ATA TAT GTT GTT GGA GGT ATT GAT AAT GAA CAT AAC 1935
631 Leu Asn Gln Ile Tyr Val Val Gly Gly Ile Asp Asn Glu His Asn 645

1936 ATA TTA GAT TCC GTT GAA CAA TAT CAA CCA TTT AAT AAA AGA TGG 1980
646 Ile Leu Asp Ser Val Glu Gln Tyr Gln Pro Phe Asn Lys Arg Trp 660

>18_K13_Sample 1

1 ATG GAA GGA GAA AAA GTA AAA ACA AAA GCA AAT AGT ATC TCG AAT 45
1 Met Glu Gly Glu Lys Val Lys Thr Lys Ala Asn Ser Ile Ser Asn 15

46 TTT TCT ATG ACG TAT GAT AGG GAA TCT GGT GGT AAC AGC AAT AGT 90
16 Phe Ser Met Thr Tyr Asp Arg Glu Ser Gly Gly Asn Ser Asn Ser 30

91 GAT GAT AAA AGC GGA AGT AGT AGC GAG AAT GAT TCT AAT TCA TTT 135
31 Asp Asp Lys Ser Gly Ser Ser Ser Glu Asn Asp Ser Asn Ser Phe 45

136 ATG AAT CTA ACT AGT GAT AAA AAT GAG AAA ACG GAA AAT AAT AGT 180
46 Met Asn Leu Thr Ser Asp Lys Asn Glu Lys Thr Glu Asn Asn Ser 60

181 TTC CTT TTA AAT AAT AGT AGT TAT GGA AAT GTT AAA GAT AGC CTA 225
61 Phe Leu Leu Asn Asn Ser Ser Tyr Gly Asn Val Lys Asp Ser Leu 75

226 TTA GAA TCC ATT GAT ATG AGT GTA TTA GAT TCG AAC TTT GAT AGT 270
76 Leu Glu Ser Ile Asp Met Ser Val Leu Asp Ser Asn Phe Asp Ser 90

271 AAA AAA GAT TTT TTA CCA AGT AAT TTA TCA AGA ACA TTT AAT AAT 315
91 Lys Lys Asp Phe Leu Pro Ser Asn Leu Ser Arg Thr Phe Asn Asn 105

316 ATG TCT AAA GAT AAT ATA GGA AAT AAA TAT TTA AAT AAA TTG TTA 360
106 Met Ser Lys Asp Asn Ile Gly Asn Lys Tyr Leu Asn Lys Leu Leu 120

361 AAT AAA AAA AAA GAT ACT ATT ACA AAT GAA AAT AAT AAT ATT AAT 405
121 Asn Lys Lys Lys Asp Thr Ile Thr Asn Glu Asn Asn Asn Ile Asn 135

406 CAT AAT AAT AAT AAT AAT AAT CTG ACA GCA AAT AAT ATA ACT AAT 450
136 His Asn Asn Asn Asn Asn Asn Leu Thr Ala Asn Asn Ile Thr Asn 150

451 AAT CTT ATT AAT AAT AAT ATG AAT TCT CCA TCA ATT ATG AAT ACC 495
151 Asn Leu Ile Asn Asn Asn Met Asn Ser Pro Ser Ile Met Asn Thr 165

496 AAC AAA AAA GAG AAT TTT TTA GAT GCA GCA AAT CTT ATA AAT GAT 540
166 Asn Lys Lys Glu Asn Phe Leu Asp Ala Ala Asn Leu Ile Asn Asp 180

541 GAT TCT GGA TTA AAC AAT TTA AAA AAA TTT TCA ACT GTA AAT AAT 585
181 Asp Ser Gly Leu Asn Asn Leu Lys Lys Phe Ser Thr Val Asn Asn 195

586 GTA AAT GAT ACT TAT GAA AAG AAA ATT ATT GAA ACG GAA TTA AGT 630
196 Val Asn Asp Thr Tyr Glu Lys Lys Ile Ile Glu Thr Glu Leu Ser 210

631 GAT GCT AGT GAT TTT GAA AAT ATG GTA GGT GAT TTA AGA ATT ACA 675
211 Asp Ala Ser Asp Phe Glu Asn Met Val Gly Asp Leu Arg Ile Thr 225

676 TTT ATT AAT TGG TTA AAA AAG ACA CAA ATG AAT TTT ATT CGA GAA 720
226 Phe Ile Asn Trp Leu Lys Lys Thr Gln Met Asn Phe Ile Arg Glu 240

721 AAA GAT AAA TTA TTT AAA GAT AAG AAA GAA CTA GAA ATG GAA AGA 765
241 Lys Asp Lys Leu Phe Lys Asp Lys Lys Glu Leu Glu Met Glu Arg 255

766 GTA CGA TTG TAC AAA GAA TTA GAA AAC CGT AAA AAT ATT GAA GAA 810
256 Val Arg Leu Tyr Lys Glu Leu Glu Asn Arg Lys Asn Ile Glu Glu 270

811 CAG AAA TTA CAT GAT GAA AGA AAG AAA TTA GAT ATT GAT ATA TCT 855
271 Gln Lys Leu His Asp Glu Arg Lys Lys Leu Asp Ile Asp Ile Ser 285

856 AAT GGT TAT AAA CAA ATA AAA AAA GAA AAA GAA GAA CAT AGG AAA 900
286 Asn Gly Tyr Lys Gln Ile Lys Lys Glu Lys Glu Glu His Arg Lys 300

901 CGA TTT GAT GAA GAA AGA TTA AGA TTT TTA CAA GAA ATC GAT AAA 945
301 Arg Phe Asp Glu Glu Arg Leu Arg Phe Leu Gln Glu Ile Asp Lys 315

946 ATT AAA TTA GTA TTA TAT TTA GAA AAA GAA AAA TAT TAT CAA GAA 990
316 Ile Lys Leu Val Leu Tyr Leu Glu Lys Glu Lys Tyr Tyr Gln Glu 330

991 TAT AAA AAT TTT GAG AAT GAT AAA AAA AAA ATT GTT GAT GCA AAT 1035
331 Tyr Lys Asn Phe Glu Asn Asp Lys Lys Lys Ile Val Asp Ala Asn 345

1036 ATT GCT ACT GAA ACT ATG ATT GAT ATT AAT GTT GGT GGA GCT ATT 1080
346 Ile Ala Thr Glu Thr Met Ile Asp Ile Asn Val Gly Gly Ala Ile 360

1081 TTT GAA ACA TCT AGA CAT ACC TTA ACA CAA CAA AAA GAT TCA TTT 1125
361 Phe Glu Thr Ser Arg His Thr Leu Thr Gln Gln Lys Asp Ser Phe 375

1126 ATA GAG AAA TTA TTA AGT GGA AGA CAT CAT GTA ACC AGA GAT AAA 1170
376 Ile Glu Lys Leu Leu Ser Gly Arg His His Val Thr Arg Asp Lys 390

1171 CAA GGA AGA ATA TTC TTA GAT AGG GAT AGT GAG TTA TTT AGA ATT 1215
391 Gln Gly Arg Ile Phe Leu Asp Arg Asp Ser Glu Leu Phe Arg Ile 405

1216 ATA CTT AAC TTC TTA AGA AAT CCG TTA ACT ATA CCC ATA CCA AAA 1260
406 Ile Leu Asn Phe Leu Arg Asn Pro Leu Thr Ile Pro Ile Pro Lys 420

1261 GAT TTA AGT GAA AGT GAA GCC TTG TTG AAA GAA GCA GAA TTT TAT 1305
421 Asp Leu Ser Glu Ser Glu Ala Leu Leu Lys Glu Ala Glu Phe Tyr 435

1306 GGT ATT AAA TTT TTA CCA TTC CCA TTA GTA TTT TGT ATA GGT GGA 1350
436 Gly Ile Lys Phe Leu Pro Phe Pro Leu Val Phe Cys Ile Gly Gly 450

1351 TTT GAT GGT GTA GAA TAT TTA AAT TCG ATG GAA TTA TTA GAT ATT 1395
451 Phe Asp Gly Val Glu Tyr Leu Asn Ser Met Glu Leu Leu Asp Ile 465

1396 AGT CAA CAA TGC TGG CGT ATG TGT ACA CCT ATG TCT ACC AAA AAA 1440
466 Ser Gln Gln Cys Trp Arg Met Cys Thr Pro Met Ser Thr Lys Lys 480

1441 GCT TAT TTT GGA AGT GCT GTA TTG AAT AAT TTC TTA TAC GTT TTT 1485
481 Ala Tyr Phe Gly Ser Ala Val Leu Asn Asn Phe Leu Tyr Val Phe 495

1486 GGT GGT AAT AAC TAT GAT TAT AAG GCT TTA TTT GAA ACT **GAA** GTG 1530
496 Gly Gly Asn Asn Tyr Asp Tyr Lys Ala Leu Phe Glu Thr **Glu** Val 510

1531 TAT GAT CGT TTA AGA GAT GTA TGG TAT GTT TCA AGT AAT TTA AAT 1575
511 Tyr Asp Arg Leu Arg Asp Val Trp Tyr Val Ser Ser Asn Leu Asn 525

1576 ATA CCT AGA AGA AAT AAT TGT GGT GTT ACG TCA AAT GGT AGA ATT 1620
526 Ile Pro Arg Arg Asn Asn Cys Gly Val Thr Ser Asn Gly Arg Ile 540

1621 TAT TGT ATT GGG GGA TAT GAT GGC TCT TCT ATT ATA CCG AAT GTA 1665
541 Tyr Cys Ile Gly Gly Tyr Asp Gly Ser Ser Ile Ile Pro Asn Val 555

1666 GAA GCA TAT GAT CAT CGT ATG AAA GCA TGG GTA GAG GTG GCA CCT 1710
556 Glu Ala Tyr Asp His Arg Met Lys Ala Trp Val Glu Val Ala Pro 570

1711 TTG AAT ACC CCT AGA TCA TCA GCT ATG TGT GTT GCT TTT GAT AAT 1755
571 Leu Asn Thr Pro Arg Ser Ser Ala Met Cys Val Ala Phe Asp Asn 585

1756 AAA ATT TAT GTC ATT GGT GGA ACT AAT GGT GAG AGG TTA AAT TCT 1800
586 Lys Ile Tyr Val Ile Gly Gly Thr Asn Gly Glu Arg Leu Asn Ser 600

1801 ATT GAA GTA TAA GAA GAA AAA ATG AAT AAA TGG GAA CAA TTT CCA 1845
601 Ile Glu Val End Glu Glu Lys Met Asn Lys Trp Glu Gln Phe Pro 615

1846 TAT GCC TTA TTA GAA GCT AGA AGT TCA GGA GCA GCT TTT AAT TAC 1890

```
616   Tyr Ala Leu Leu Glu Ala Arg Ser Ser Gly Ala Ala Phe Asn Tyr   630

1891  CTT AAT CAA ATA TAT GTT GTT GGA GGT ATT   1920
631   Leu Asn Gln Ile Tyr Val Val Gly Gly Ile   640
```

>19_K13_Sample 2

```
1     ATG GAA GGA GAA AAA GTA AAA ACA AAA GCA AAT AGT ATC TCG AAT   45
1     Met Glu Gly Glu Lys Val Lys Thr Lys Ala Asn Ser Ile Ser Asn   15

46    TTT TCT ATG ACG TAT GAT AGG GAA TCT GGT GGT AAC AGC AAT AGT   90
16    Phe Ser Met Thr Tyr Asp Arg Glu Ser Gly Gly Asn Ser Asn Ser   30

91    GAT GAT AAA AGC GGA AGT AGT AGC GAG AAT GAT TCT AAT TCA TTT   135
31    Asp Asp Lys Ser Gly Ser Ser Ser Glu Asn Asp Ser Asn Ser Phe   45

136   ATG AAT CTA ACT AGT GAT AAA AAT GAG AAA ACG GAA AAT AAT AGT   180
46    Met Asn Leu Thr Ser Asp Lys Asn Glu Lys Thr Glu Asn Asn Ser   60

181   TTC CTT TTA AAT AAT AGT AGT TAT GGA AAT GTT AAA GAT AGC CTA   225
61    Phe Leu Leu Asn Asn Ser Ser Tyr Gly Asn Val Lys Asp Ser Leu   75

226   TTA GAA TCC ATT GAT ATG AGT GTA TTA GAT TCG AAC TTT GAT AGT   270
76    Leu Glu Ser Ile Asp Met Ser Val Leu Asp Ser Asn Phe Asp Ser   90

271   AAA AAA GAT TTT TTA CCA AGT AAT TTA TCA AGA ACA TTT AAT AAT   315
91    Lys Lys Asp Phe Leu Pro Ser Asn Leu Ser Arg Thr Phe Asn Asn   105

316   ATG TCT AAA GAT AAT ATA GGA AAT AAA TAT TTA AAT AAA TTG TTA   360
106   Met Ser Lys Asp Asn Ile Gly Asn Lys Tyr Leu Asn Lys Leu Leu   120

361   AAT AAA AAA AAA GAT ACT ATT ACA AAT GAA AAT AAT AAT ATT AAT   405
121   Asn Lys Lys Lys Asp Thr Ile Thr Asn Glu Asn Asn Asn Ile Asn   135

406   CAT AAT AAT AAT AAT AAT AAT CTG ACA GCA AAT AAT ATA ACT AAT   450
136   His Asn Asn Asn Asn Asn Asn Leu Thr Ala Asn Asn Ile Thr Asn   150

451   AAT CTT ATT AAT AAT AAT ATG AAT TCT CCA TCA ATT ATG AAT ACC   495
151   Asn Leu Ile Asn Asn Asn Met Asn Ser Pro Ser Ile Met Asn Thr   165

496   AAC AAA AAA GAG AAT TTT TTA GAT GCA GCA AAT CTT ATA AAT GAT   540
166   Asn Lys Lys Glu Asn Phe Leu Asp Ala Ala Asn Leu Ile Asn Asp   180

541   GAT TCT GGA TTA AAC AAT TTA AAA AAA TTT TCA ACT GTA AAT AAT   585
181   Asp Ser Gly Leu Asn Asn Leu Lys Lys Phe Ser Thr Val Asn Asn   195

586   GTA AAT GAT ACT TAT GAA AAG AAA ATT ATT GAA ACG GAA TTA AGT   630
196   Val Asn Asp Thr Tyr Glu Lys Lys Ile Ile Glu Thr Glu Leu Ser   210

631   GAT GCT AGT GAT TTT GAA AAT ATG GTA GGT GAT TTA AGA ATT ACA   675
211   Asp Ala Ser Asp Phe Glu Asn Met Val Gly Asp Leu Arg Ile Thr   225

676   TTT ATT AAT TGG TTA AAA AAG ACA CAA ATG AAT TTT ATT CGA GAA   720
```

226 Phe Ile Asn Trp Leu Lys Lys Thr Gln Met Asn Phe Ile Arg Glu 240

721 AAA GAT AAA TTA TTT AAA GAT AAG AAA GAA CTA GAA ATG GAA AGA 765
241 Lys Asp Lys Leu Phe Lys Asp Lys Lys Glu Leu Glu Met Glu Arg 255

766 GTA CGA TTG TAC AAA GAA TTA GAA AAC CGT AAA AAT ATT GAA GAA 810
256 Val Arg Leu Tyr Lys Glu Leu Glu Asn Arg Lys Asn Ile Glu Glu 270

811 CAG AAA TTA CAT GAT GAA AGA AAG AAA TTA GAT ATT GAT ATA TCT 855
271 Gln Lys Leu His Asp Glu Arg Lys Lys Leu Asp Ile Asp Ile Ser 285

856 AAT GGT TAT AAA CAA ATA AAA AAA GAA AAA GAA GAA CAT AGG AAA 900
286 Asn Gly Tyr Lys Gln Ile Lys Lys Glu Lys Glu Glu His Arg Lys 300

901 CGA TTT GAT GAA GAA AGA TTA AGA TTT TTA CAA GAA ATC GAT AAA 945
301 Arg Phe Asp Glu Glu Arg Leu Arg Phe Leu Gln Glu Ile Asp Lys 315

946 ATT AAA TTA GTA TTA TAT TTA GAA AAA GAA AAA TAT TAT CAA GAA 990
316 Ile Lys Leu Val Leu Tyr Leu Glu Lys Glu Lys Tyr Tyr Gln Glu 330

991 TAT AAA AAT TTT GAG AAT GAT AAA AAA AAA ATT GTT GAT GCA AAT 1035
331 Tyr Lys Asn Phe Glu Asn Asp Lys Lys Lys Ile Val Asp Ala Asn 345

1036 ATT GCT ACT GAA ACT ATG ATT GAT ATT AAT GTT GGT GGA GCT ATT 1080
346 Ile Ala Thr Glu Thr Met Ile Asp Ile Asn Val Gly Gly Ala Ile 360

1081 TTT GAA ACA TCT AGA CAT ACC TTA ACA CAA CAA AAA GAT TCA TTT 1125
361 Phe Glu Thr Ser Arg His Thr Leu Thr Gln Gln Lys Asp Ser Phe 375

1126 ATA GAG AAA TTA TTA AGT GGA AGA CAT CAT GTA ACC AGA GAT AAA 1170
376 Ile Glu Lys Leu Leu Ser Gly Arg His His Val Thr Arg Asp Lys 390

1171 CAA GGA AGA ATA TTC TTA GAT AGG GAT AGT GAG TTA TTT AGA ATT 1215
391 Gln Gly Arg Ile Phe Leu Asp Arg Asp Ser Glu Leu Phe Arg Ile 405

1216 ATA CTT AAC TTC TTA AGA AAT CCG TTA ACT ATA CCC ATA CCA AAA 1260
406 Ile Leu Asn Phe Leu Arg Asn Pro Leu Thr Ile Pro Ile Pro Lys 420

1261 GAT TTA AGT GAA AGT GAA GCC TTG TTG AAA GAA GCA GAA TTT TAT 1305
421 Asp Leu Ser Glu Ser Glu Ala Leu Leu Lys Glu Ala Glu Phe Tyr 435

1306 GGT ATT AAA TTT TTA CCA TTC CCA TTA GTA TTT TGT ATA GGT GGA 1350
436 Gly Ile Lys Phe Leu Pro Phe Pro Leu Val Phe Cys Ile Gly Gly 450

1351 TTT GAT GGT GTA GAA TAT TTA AAT TCG ATG GAA TTA TTA GAT ATT 1395
451 Phe Asp Gly Val Glu Tyr Leu Asn Ser Met Glu Leu Leu Asp Ile 465

1396 AGT CAA CAA TGC TGG CGT ATG TGT ACA CCT ATG TCT ACC AAA AAA 1440
466 Ser Gln Gln Cys Trp Arg Met Cys Thr Pro Met Ser Thr Lys Lys 480

1441 GCT TAT TTT GGA AGT GCT GTA TTG AAT AAT TTC TTA TAC GTT TTT 1485
481 Ala Tyr Phe Gly Ser Ala Val Leu Asn Asn Phe Leu Tyr Val Phe 495

1486 GGT GGT AAT AAC TAT GAT TAT AAG GCT TTA TTT GAA ACT GAG GTG 1530
496 Gly Gly Asn Asn Tyr Asp Tyr Lys Ala Leu Phe Glu Thr Glu Val 510

1531 TAT GAT CGT TTA AGA GAT GTA TGG TAT GTT TCA AGT AAT TTA AAT 1575
511 Tyr Asp Arg Leu Arg Asp Val Trp Tyr Val Ser Ser Asn Leu Asn 525

1576 ATA CCT AGA AGA AAT AAT TGT **TGT** GTT ACG TCA AAT GGT AGA ATT 1620
526 Ile Pro Arg Arg Asn Asn Cys **Cys** Val Thr Ser Asn Gly Arg Ile 540

1621 TAT TGT ATT GGG GGA TAT GAT GGC TCT TCT ATT ATA CCG AAT GTA 1665
541 Tyr Cys Ile Gly Gly Tyr Asp Gly Ser Ser Ile Ile Pro Asn Val 555

1666 GAA GCA TAT GAT CAT CGT ATG AAA GCA TGG GTA GAG GTG GCA CCT 1710
556 Glu Ala Tyr Asp His Arg Met Lys Ala Trp Val Glu Val Ala Pro 570

1711 TTG AAT ACC CCT AGA TCA TCA GCT ATG TGT GTT GCT TTT GAT AAT 1755
571 Leu Asn Thr Pro Arg Ser Ser Ala Met Cys Val Ala Phe Asp Asn 585

1756 AAA ATT TAT GTC ATT GGT GGA ACT AAT GGT GAG AGA TTA AAT TCT 1800
586 Lys Ile Tyr Val Ile Gly Gly Thr Asn Gly Glu Arg Leu Asn Ser 600

1801 ATT GAA GTA TAT GAA GAA AAA ATG AAT AAA TGG GAA CAA TTT CCA 1845
601 Ile Glu Val Tyr Glu Glu Lys Met Asn Lys Trp Glu Gln Phe Pro 615

1846 TAT GCC TTA TTA GAA GCT AGA AGT TCA GGA GCA GCT TTT AAT TAC 1890
616 Tyr Ala Leu Leu Glu Ala Arg Ser Ser Gly Ala Ala Phe Asn Tyr 630

1891 CTT AAT CAA ATA TAT GTT GTT GGA GGT ATT 1920
631 Leu Asn Gln Ile Tyr Val Val Gly Gly Ile 640

>22_K13_Sample 3

1 ATG GAA GGA GAA AAA GTA AAA ACA AAA GCA AAT AGT ATC TCG AAT 45
1 Met Glu Gly Glu Lys Val Lys Thr Lys Ala Asn Ser Ile Ser Asn 15

46 TTT TCT ATG ACG TAT GAT AGG GAA TCT GGT GGT AAC AGC AAT AGT 90
16 Phe Ser Met Thr Tyr Asp Arg Glu Ser Gly Gly Asn Ser Asn Ser 30

91 GAT GAT AAA AGC GGA AGT AGT AGC GAG AAT GAT TCT AAT TCA TTT 135
31 Asp Asp Lys Ser Gly Ser Ser Ser Glu Asn Asp Ser Asn Ser Phe 45

136 ATG AAT CTA ACT AGT GAT AAA AAT GAG AAA ACG GAA AAT AAT AGT 180
46 Met Asn Leu Thr Ser Asp Lys Asn Glu Lys Thr Glu Asn Asn Ser 60

181 TTC CTT TTA AAT AAT AGT AGT TAT GGA AAT GTT AAA GAT AGC CTA 225
61 Phe Leu Leu Asn Asn Ser Ser Tyr Gly Asn Val Lys Asp Ser Leu 75

226 TTA GAA TCC ATT GAT ATG AGT GTA TTA GAT TCG AAC TTT GAT AGT 270
76 Leu Glu Ser Ile Asp Met Ser Val Leu Asp Ser Asn Phe Asp Ser 90

271 AAA AAA GAT TTT TTA CCA AGT AAT TTA TCA AGA ACA TTT AAT AAT 315
91 Lys Lys Asp Phe Leu Pro Ser Asn Leu Ser Arg Thr Phe Asn Asn 105

```
316   ATG TCT AAA GAT AAT ATA GGA AAT AAA TAT TTA AAT AAA TTG TTA   360
106   Met Ser Lys Asp Asn Ile Gly Asn Lys Tyr Leu Asn Lys Leu Leu   120

361   AAT AAA AAA AAA GAT ACT ATT ACA AAT GAA AAT AAT AAT ATT AAT   405
121   Asn Lys Lys Lys Asp Thr Ile Thr Asn Glu Asn Asn Asn Ile Asn   135

406   CAT AAT AAT AAT AAT AAT AAT CTG ACA GCA AAT AAT ATA ACT AAT   450
136   His Asn Asn Asn Asn Asn Asn Leu Thr Ala Asn Asn Ile Thr Asn   150

451   AAT CTT ATT AAT AAT AAT ATG AAT TCT CCA TCA ATT ATG AAT ACC   495
151   Asn Leu Ile Asn Asn Asn Met Asn Ser Pro Ser Ile Met Asn Thr   165

496   AAC AAA AAA GAG AAT TTT TTA GAT GCA GCA AAT CTT ATA AAT GAT   540
166   Asn Lys Lys Glu Asn Phe Leu Asp Ala Ala Asn Leu Ile Asn Asp   180

541   GAT TCT GGA TTA AAC AAT TTA AAA AAA TTT TCA ACT GTA AAT AAT   585
181   Asp Ser Gly Leu Asn Asn Leu Lys Lys Phe Ser Thr Val Asn Asn   195

586   GTA AAT GAT ACT TAT GAA AAG AAA ATT ATT GAA ACG GAA TTA AGT   630
196   Val Asn Asp Thr Tyr Glu Lys Lys Ile Ile Glu Thr Glu Leu Ser   210

631   GAT GCT AGT GAT TTT GAA AAT ATG GTA GGT GAT TTA AGA ATT ACA   675
211   Asp Ala Ser Asp Phe Glu Asn Met Val Gly Asp Leu Arg Ile Thr   225

676   TTT ATT AAT TGG TTA AAA AAG ACA CAA ATG AAT TTT ATT CGA GAA   720
226   Phe Ile Asn Trp Leu Lys Lys Thr Gln Met Asn Phe Ile Arg Glu   240

721   AAA GAT AAA TTA TTT AAA GAT AAG AAA GAA CTA GAA ATG GAA AGA   765
241   Lys Asp Lys Leu Phe Lys Asp Lys Lys Glu Leu Glu Met Glu Arg   255

766   GTA CGA TTG TAC AAA GAA TTA GAA AAC CGT AAA AAT ATT GAA GAA   810
256   Val Arg Leu Tyr Lys Glu Leu Glu Asn Arg Lys Asn Ile Glu Glu   270

811   CAG AAA TTA CAT GAT GAA AGA AAG AAA TTA GAT ATT GAT ATA TCT   855
271   Gln Lys Leu His Asp Glu Arg Lys Lys Leu Asp Ile Asp Ile Ser   285

856   AAT GGT TAT AAA CAA ATA AAA AAA GAA AAA GAA GAA CAT AGG AAA   900
286   Asn Gly Tyr Lys Gln Ile Lys Lys Glu Lys Glu Glu His Arg Lys   300

901   CGA TTT GAT GAA GAA AGA TTA AGA TTT TTA CAA GAA ATC GAT AAA   945
301   Arg Phe Asp Glu Glu Arg Leu Arg Phe Leu Gln Glu Ile Asp Lys   315

946   ATT AAA TTA GTA TTA TAT TTA GAA AAA GAA AAA TAT TAT CAA GAA   990
316   Ile Lys Leu Val Leu Tyr Leu Glu Lys Glu Lys Tyr Tyr Gln Glu   330

991   TAT AAA AAT TTT GAG AAT GAT AAA AAA AAA ATT GTT GAT GCA AAT   1035
331   Tyr Lys Asn Phe Glu Asn Asp Lys Lys Lys Ile Val Asp Ala Asn   345

1036  ATT GCT ACT GAA ACT ATG ATT GAT ATT AAT GTT GGT GGA GCT ATT   1080
346   Ile Ala Thr Glu Thr Met Ile Asp Ile Asn Val Gly Gly Ala Ile   360

1081  TTT GAA ACA TCT AGA CAT ACC TTA ACA CAA CAA AAA GAT TCA TTT   1125
361   Phe Glu Thr Ser Arg His Thr Leu Thr Gln Gln Lys Asp Ser Phe   375
```

1126 ATA GAG AAA TTA TTA AGT GGA AGA CAT CAT GTA ACC AGA GAT AAA 1170
376 Ile Glu Lys Leu Leu Ser Gly Arg His His Val Thr Arg Asp Lys 390

1171 CAA GGA AGA ATA TTC TTA GAT AGG GAT AGT GAG TTA TTT AGA ATT 1215
391 Gln Gly Arg Ile Phe Leu Asp Arg Asp Ser Glu Leu Phe Arg Ile 405

1216 ATA CTT AAC TTC TTA AGA AAT CCG TTA ACT ATA CCC ATA CCA AAA 1260
406 Ile Leu Asn Phe Leu Arg Asn Pro Leu Thr Ile Pro Ile Pro Lys 420

1261 GAT TTA AGT GAA AGT GAA GCC TTG TTG AAA GAA GCA GAA TTT TAT 1305
421 Asp Leu Ser Glu Ser Glu Ala Leu Leu Lys Glu Ala Glu Phe Tyr 435

1306 GGT ATT AAA TTT TTA CCA TTC CCA TTA GTA TTT TGT ATA GGT GGA 1350
436 Gly Ile Lys Phe Leu Pro Phe Pro Leu Val Phe Cys Ile Gly Gly 450

1351 TTT GAT GGT GTA GAA TAT TTA AAT TCG ATG GAA TTA TTA GAT ATT 1395
451 Phe Asp Gly Val Glu Tyr Leu Asn Ser Met Glu Leu Leu Asp Ile 465

1396 AGT CAA CAA TGC TGG CGT ATG TGT ACA CCT ATG TCT ACC AAA AAA 1440
466 Ser Gln Gln Cys Trp Arg Met Cys Thr Pro Met Ser Thr Lys Lys 480

1441 GCT TAT TTT GGA AGT GCT GTA TTG AAT AAT TTC TTA TAC GTT TTT 1485
481 Ala Tyr Phe Gly Ser Ala Val Leu Asn Asn Phe Leu Tyr Val Phe 495

1486 GGT GGT AAT AAC TAT GAT TAT AAG GCT TTA TTT GAA ACT **GAA** GTG 1530
496 Gly Gly Asn Asn Tyr Asp Tyr Lys Ala Leu Phe Glu Thr **Glu** Val 510

1531 TAT GAT CGT TTA AGA GAT GTA TGG TAT GTT TCA AGT AAT TTA AAT 1575
511 Tyr Asp Arg Leu Arg Asp Val Trp Tyr Val Ser Ser Asn Leu Asn 525

1576 ATA CCT AGA AGA AAT AAT TGT GGT GTT ACG TCA AAT GGT AGA ATT 1620
526 Ile Pro Arg Arg Asn Asn Cys Gly Val Thr Ser Asn Gly Arg Ile 540

1621 TAT TGT ATT GGG GGA TAT GAT GGC TCT TCT ATT ATA CCG AAT GTA 1665
541 Tyr Cys Ile Gly Gly Tyr Asp Gly Ser Ser Ile Ile Pro Asn Val 555

1666 GAA GCA TAT GAT CAT CGT ATG AAA GCA TGG GTA GAG GTG GCA CCT 1710
556 Glu Ala Tyr Asp His Arg Met Lys Ala Trp Val Glu Val Ala Pro 570

1711 TTG AAT ACC CCT AGA TCA TCA GCT ATG TGT GTT GCT TTT GAT AAT 1755
571 Leu Asn Thr Pro Arg Ser Ser Ala Met Cys Val Ala Phe Asp Asn 585

1756 AAA ATT TAT GTC ATT GGT GGA ACT AAT GGT GAG AGG TTA AAT TCT 1800
586 Lys Ile Tyr Val Ile Gly Gly Thr Asn Gly Glu Arg Leu Asn Ser 600

1801 ATT GAA GTA TAT GAA GAA AAA ATG AAT AAA TGG GAA CAA TTT CCA 1845
601 Ile Glu Val Tyr Glu Glu Lys Met Asn Lys Trp Glu Gln Phe Pro 615

1846 TAT GCC TTA TTA GAA GCT AGA AGT TCA GGA GCA GCT TTT AAT TAC 1890
616 Tyr Ala Leu Leu Glu Ala Arg Ser Ser Gly Ala Ala Phe Asn Tyr 630

1891 CTT AAT CAA ATA TAT GTT GTT GGA GGT ATT 1920

631 Leu Asn Gln Ile Tyr Val Val Gly Gly Ile 640

>46_K13_Sample 4

1 ATG GAA GGA GAA AAA GTA AAA ACA AAA GCA AAT AGT ATC TCG AAT 45
1 Met Glu Gly Glu Lys Val Lys Thr Lys Ala Asn Ser Ile Ser Asn 15

46 TTT TCT ATG ACG TAT GAT AGG GAA TCT GGT GGT AAC AGC AAT AGT 90
16 Phe Ser Met Thr Tyr Asp Arg Glu Ser Gly Gly Asn Ser Asn Ser 30

91 GAT GAT AAA AGC GGA AGT AGT AGC GAG AAT GAT TCT AAT TCA TTT 135
31 Asp Asp Lys Ser Gly Ser Ser Ser Glu Asn Asp Ser Asn Ser Phe 45

136 ATG AAT CTA ACT AGT GAT AAA AAT GAG AAA ACG GAA AAT AAT AGT 180
46 Met Asn Leu Thr Ser Asp Lys Asn Glu Lys Thr Glu Asn Asn Ser 60

181 TTC CTT TTA AAT AAT AGT AGT TAT GGA AAT GTT AAA GAT AGC CTA 225
61 Phe Leu Leu Asn Asn Ser Ser Tyr Gly Asn Val Lys Asp Ser Leu 75

226 TTA GAA TCC ATT GAT ATG AGT GTA TTA GAT TCG AAC TTT GAT AGT 270
76 Leu Glu Ser Ile Asp Met Ser Val Leu Asp Ser Asn Phe Asp Ser 90

271 AAA AAA GAT TTT TTA CCA AGT AAT TTA TCA AGA ACA TTT AAT AAT 315
91 Lys Lys Asp Phe Leu Pro Ser Asn Leu Ser Arg Thr Phe Asn Asn 105

316 ATG TCT AAA GAT AAT ATA GGA AAT AAA TAT TTA AAT AAA TTG TTA 360
106 Met Ser Lys Asp Asn Ile Gly Asn Lys Tyr Leu Asn Lys Leu Leu 120

361 AAT AAA AAA AAA GAT ACT ATT ACA AAT GAA AAT AAT AAT ATT AAT 405
121 Asn Lys Lys Lys Asp Thr Ile Thr Asn Glu Asn Asn Asn Ile Asn 135

406 CAT AAT AAT AAT AAT AAT AAT CTG ACA GCA AAT AAT ATA ACT AAT 450
136 His Asn Asn Asn Asn Asn Asn Leu Thr Ala Asn Asn Ile Thr Asn 150

451 AAT CTT ATT AAT AAT AAT ATG AAT TCT CCA TCA ATT ATG AAT ACC 495
151 Asn Leu Ile Asn Asn Asn Met Asn Ser Pro Ser Ile Met Asn Thr 165

496 AAC AAA AAA GAG AAT TTT TTA GAT GCA GCA AAT CTT ATA AAT GAT 540
166 Asn Lys Lys Glu Asn Phe Leu Asp Ala Ala Asn Leu Ile Asn Asp 180

541 GAT TCT GGA TTA AAC AAT TTA AAA AAA TTT TCA ACT GTA AAT AAT 585
181 Asp Ser Gly Leu Asn Asn Leu Lys Lys Phe Ser Thr Val Asn Asn 195

586 GTA AAT GAT ACT TAT GAA AAG AAA ATT ATT GAA ACG GAA TTA AGT 630
196 Val Asn Asp Thr Tyr Glu Lys Lys Ile Ile Glu Thr Glu Leu Ser 210

631 GAT GCT AGT GAT TTT GAA AAT ATG GTA GGT GAT TTA AGA ATT ACA 675
211 Asp Ala Ser Asp Phe Glu Asn Met Val Gly Asp Leu Arg Ile Thr 225

676 TTT ATT AAT TGG TTA AAA AAG ACA CAA ATG AAT TTT ATT CGA GAA 720
226 Phe Ile Asn Trp Leu Lys Lys Thr Gln Met Asn Phe Ile Arg Glu 240

721 AAA GAT AAA TTA TTT AAA GAT AAG AAA GAA CTA GAA ATG GAA AGA 765

241 Lys Asp Lys Leu Phe Lys Asp Lys Lys Glu Leu Glu Met Glu Arg 255

766 GTA CGA TTG TAC AAA GAA TTA GAA AAC CGT AAA AAT ATT GAA GAA 810
256 Val Arg Leu Tyr Lys Glu Leu Glu Asn Arg Lys Asn Ile Glu Glu 270

811 CAG AAA TTA CAT GAT GAA AGA AAG AAA TTA GAT ATT GAT ATA TCT 855
271 Gln Lys Leu His Asp Glu Arg Lys Lys Leu Asp Ile Asp Ile Ser 285

856 AAT GGT TAT AAA CAA ATA AAA AAA GAA AAA GAA GAA CAT AGG AAA 900
286 Asn Gly Tyr Lys Gln Ile Lys Lys Glu Lys Glu Glu His Arg Lys 300

901 CGA TTT GAT GAA GAA AGA TTA AGA TTT TTA CAA GAA ATC GAT AAA 945
301 Arg Phe Asp Glu Glu Arg Leu Arg Phe Leu Gln Glu Ile Asp Lys 315

946 ATT AAA TTA GTA TTA TAT TTA GAA AAA GAA AAA TAT TAT CAA GAA 990
316 Ile Lys Leu Val Leu Tyr Leu Glu Lys Glu Lys Tyr Tyr Gln Glu 330

991 TAT AAA AAT TTT GAG AAT GAT AAA AAA AAA ATT GTT GAT GCA AAT 1035
331 Tyr Lys Asn Phe Glu Asn Asp Lys Lys Lys Ile Val Asp Ala Asn 345

1036 ATT GCT ACT GAA ACT ATG ATT GAT ATT AAT GTT GGT GGA GCT ATT 1080
346 Ile Ala Thr Glu Thr Met Ile Asp Ile Asn Val Gly Gly Ala Ile 360

1081 TTT GAA ACA TCT AGA CAT ACC TTA ACA CAA CAA AAA GAT TCA TTT 1125
361 Phe Glu Thr Ser Arg His Thr Leu Thr Gln Gln Lys Asp Ser Phe 375

1126 ATA GAG AAA TTA TTA AGT GGA AGA CAT CAT GTA ACC AGA GAT AAA 1170
376 Ile Glu Lys Leu Leu Ser Gly Arg His His Val Thr Arg Asp Lys 390

1171 CAA GGA AGA ATA TTC TTA GAT AGG GAT AGT GAG TTA TTT AGA ATT 1215
391 Gln Gly Arg Ile Phe Leu Asp Arg Asp Ser Glu Leu Phe Arg Ile 405

1216 ATA CTT AAC TTC TTA AGA AAT CCG TTA ACT ATA CCC ATA CCA AAA 1260
406 Ile Leu Asn Phe Leu Arg Asn Pro Leu Thr Ile Pro Ile Pro Lys 420

1261 GAT TTA AGT GAA AGT GAA GCC TTG TTG AAA GAA GCA GAA TTT TAT 1305
421 Asp Leu Ser Glu Ser Glu Ala Leu Leu Lys Glu Ala Glu Phe Tyr 435

1306 GGT ATT AAA TTT TTA CCA TTC CCA TTA GTA TTT TGT ATA GGT GGA 1350
436 Gly Ile Lys Phe Leu Pro Phe Pro Leu Val Phe Cys Ile Gly Gly 450

1351 TTT GAT GGT GTA GAA TAT TTA AAT TCG ATG GAA TTA TTA GAT ATT 1395
451 Phe Asp Gly Val Glu Tyr Leu Asn Ser Met Glu Leu Leu Asp Ile 465

1396 AGT CAA CAA TGC TGG CGT ATG TGT ACA CCT ATG TCT ACC AAA AAA 1440
466 Ser Gln Gln Cys Trp Arg Met Cys Thr Pro Met Ser Thr Lys Lys 480

1441 GCT TAT TTT GGA AGT GCT GTA TTG AAT AAT TTC TTA TAC GTT TTT 1485
481 Ala Tyr Phe Gly Ser Ala Val Leu Asn Asn Phe Leu Tyr Val Phe 495

1486 GGT GGT AAT AAC TAT GAT TAT AAG GCT TTA TTT GAA ACT GAG GTG 1530
496 Gly Gly Asn Asn Tyr Asp Tyr Lys Ala Leu Phe Glu Thr Glu Val 510

```
1531   TAT GAT CGT TTA AGA GAT GTA TGG TAT GTT TCA AGG AAT TTA AAT   1575
511    Tyr Asp Arg Leu Arg Asp Val Trp Tyr Val Ser Arg Asn Leu Asn   525

1576   ATA CCT AGA AGA AAT AAT TGT GGT GTT ACG TCA AAT GGT AGA ATT   1620
526    Ile Pro Arg Arg Asn Asn Cys Gly Val Thr Ser Asn Gly Arg Ile   540

1621   TAT TGT ATT GGG GGA TAT GAT GGC TCT TCT ATT ATA CCG AAT GTA   1665
541    Tyr Cys Ile Gly Gly Tyr Asp Gly Ser Ser Ile Ile Pro Asn Val   555

1666   GAA GCA TAT GAT CAT CGT ATG AAA GCA TGG GTA GAG GTG GCA CCT   1710
556    Glu Ala Tyr Asp His Arg Met Lys Ala Trp Val Glu Val Ala Pro   570

1711   TTG AAT ACC CCT AGA TCA TCA GCT ATG TGT GTT GCT TTT GAT AAT   1755
571    Leu Asn Thr Pro Arg Ser Ser Ala Met Cys Val Ala Phe Asp Asn   585

1756   AAA ATT TAT GTC ATT GGT GGA ACT AAT GGT GAG AGA TTA AAT TCT   1800
586    Lys Ile Tyr Val Ile Gly Gly Thr Asn Gly Glu Arg Leu Asn Ser   600

1801   ATT GAA GTA TAT GAA GAA AAA ATG AAT AAA TGG GAA CAA TTT CCA   1845
601    Ile Glu Val Tyr Glu Glu Lys Met Asn Lys Trp Glu Gln Phe Pro   615

1846   TAT GCC TTA TTA GAA GCT AGA AGT TCA GGA GCA GCT TTT AAT TAC   1890
616    Tyr Ala Leu Leu Glu Ala Arg Ser Ser Gly Ala Ala Phe Asn Tyr   630

1891   CTT AAT CAA ATA TAT GTT GTT AGA GGT ATT   1920
631    Leu Asn Gln Ile Tyr Val Val Arg Gly Ile   640
```

Reference Codon   Mutant Codon
